# Characteristics of noncontinuous multilevel spinal fractures: an age-group specific analysis

**DOI:** 10.1007/s00590-025-04432-z

**Published:** 2025-07-10

**Authors:** Julius Gerstmeyer, Paulina Schlehenkamp, Aileen Spieckermann, Maria Bernstorff, Mirko Aach, Emre Yilmaz, Thomas A. Schildhauer, Alexander von Glinski, Christiane Kruppa

**Affiliations:** 1 Department of General and Trauma Surgery, BG University Hospital Bergmannsheil, Bochum, Germany; 2 Department of Orthopaedic and Trauma Surgery, St. Josef Hospital Bochum, Bochum, Germany

**Keywords:** Vertebral body fracture, Multilevel fracture, Non-continuous fracture, Spine, Spine surgery

## Abstract

**Objective:**

Noncontinuous multilevel fractures (NMF) are complex injuries characterized by spinal fractures at non-adjacent levels, with reported incidence ranging from 1.6 to 17.7% depending on imaging modalities. Typically resulting from high-energy trauma such as motor vehicle accidents, NMF can also occur due to low-energy mechanisms, especially among elderly patients. We aimed to investigate clinical characteristics and fracture morphology of NMF across age groups.

**Methods:**

We retrospectively reviewed trauma patients treated for NMF at a tertiary center from 2014 to 2021. Demographics, diagnosis, trauma mechanisms, admission, and treatment data were collected. Fractures were classified by location and type. Patients were stratified into two age groups: younger (< 65 years) and older (≥ 65 years). Descriptive statistical analysis was performed.

**Results:**

Eighty-six patients (median age 59.5 ± 16.8 years; 64% male; < 65 years, n = 51; ≥ 65 years, n = 35) met inclusion criteria. ICU admission occurred in 65.1%; spinal cord injury was present in 12.8%. High-energy trauma predominated among younger patients (52.9% vs. 25.7%), whereas older patients experienced more low-energy falls (31.4% vs. 7.8%). Of 342 fractures identified, 221 occurred in younger patients; AO type A fractures and fractures in semi-rigid spinal regions were most common. Younger patients had more associated injuries. Overall, surgical treatment was performed in 76.5% of patients without age differences; 14% required revision surgery. Postoperative pneumonia affected 50%, with similar complication rates across age groups.

**Conclusion:**

Despite distinct trauma mechanisms and fracture morphologies between age groups, rates of surgical treatment and postoperative complications were comparable. Age-adapted diagnostic and management strategies for NMF may be beneficial.

## Introduction

Noncontinuous multilevel fractures (NMF) of the spine represent a complex injury pattern characterized by fractures occurring at non-adjacent spinal levels. Reported incidence rates vary widely, from 1.6 to 17.7%, depending on the imaging modality used [1–4]. Kanna et al. highlighted that spinal injuries were overlooked in up to 28.6% of trauma patients, based on whole-spine MRI scans [1]. This diagnostic variability underscores the challenge of identifying NMF, particularly among elderly patients, for whom missed injuries may lead to significant morbidity. Typically, NMF result from high-energy trauma, including motor vehicle accidents (MVA) or falls from significant heights. However, in elderly populations with lower bone mineral density, even low-energy trauma can result in substantial osteoporotic fractures [1, 5]. Prior studies have reported that the thoracolumbar region is most frequently affected in NMF, often accompanied by extraspinal injuries and spinal cord involvement [5–8]. Wang et al. demonstrated age-specific differences in NMF characteristics within a Chinese population, noting variations in spinal cord injury rates and fracture distribution patterns across age groups [5].Given recent advances in diagnostic imaging, particularly MRI, and improvements in therapeutic approaches, it remains unclear whether previous findings still accurately reflect current clinical practice. The objective of this retrospective study was to investigate the epidemiological characteristics, fracture morphology, and treatment modalities for NMF among different age groups within a contemporary patient cohort.

## Methods

We performed a retrospective chart review of all patients admitted with spinal fractures to a tertiary-level academic trauma center in Germany between 2014 and 2021. Charts were screened for the presence of NMF, defined as multiple spinal fractures separated by at least one intact vertebra. Senior spine surgeons determined indications for surgical or non-surgical management. Collected data included patient demographics, primary diagnosis, associated injuries, trauma mechanism, spinal cord injury (categorized as sensory deficits, paraplegia, or tetraplegia), admission date, and length of hospital stay. Additional data included intensive care unit (ICU) admission, complications during hospitalization, surgical duration, and the necessity for revision surgery. Surgical treatment was defined as surgery performed on at least one spinal level. Each fracture was classified 2 senior spine surgeons using CT-Scans by anatomical location and AO-Spine criteria (axial, sub axial, thoracolumbar). Locations were determined according to the Spine Instability Neoplastic Score (SINS) as junctional (O–C2, C7–T2, T11–L1, L5–S1), mobile (C3–C6, L2–L4), semi-rigid (T3–T10), or rigid (S2–S5). Complications were categorized as medical or surgical. Medical complications included multiorgan failure, pneumonia, sepsis, and pulmonary embolism. Surgical complications were defined as wound- or hardware-related events requiring revision surgery. This study was conducted with institutional ethics board approval in accordance with the Declaration of Helsinki (approval no. 2024-230-f-S).

Statistical analyses were performed using SPSS software (IBM® SPSS Statistics®, Version 29.0.2.0 (20), Armonk, NY, USA). Patients were stratified into two subgroups by age (≥ 65 years vs. < 65 years). Descriptive statistics are presented as mean with standard deviation (SD) for continuous variables or as frequencies with percentages for categorical variables. Comparisons between categorical variables were conducted using the Chi-square test. Continuous variables were analyzed with the independent-samples t-test. The significance level was set at p = 0.05.

## Results

Overall, 86 patients met inclusion criteria (mean age 59.5 ± 16.8 years), with males comprising 67.4% of the cohort. Most patients < 65 years (n = 51), with 35 patients being ≥ 65 years old. Mean age differed significantly between groups: 48.6 ± 12.4 years (< 65 years) versus 75.5 ± 6.3 years (≥ 65 years). Gender distribution also differed significantly, with males more prevalent in the < 65 years group (82.4% vs. 45.7%; p < 0.001). The overall mean length of hospital stay was 21.4 ± 28.9 days, significantly longer in the < 65 years group (26 days vs. 14 days; p = 0.043). Intensive care unit (ICU) admission occurred in 65.1% of patients. In-hospital mortality occurred exclusively in the ≥ 65 years group, yielding an overall mortality rate of 5.8%. Spinal cord injury (SCI) was present in 12.8% of cases, with sensory deficits in 7%, showing no significant difference between age groups. Ankylosing spondylitis and osteoporosis predominantly affected patients in the ≥ 65 years group (Table 1).

**Table 1:** Demographics of patients

Patient demographics	Patients < 65 N = 51	Patients ≥ 65 N = 35	Total N = 86	p-value
		N (%) or	Mean (± SD)	
Age	48.6 (± 12.4)	75.5 (± 6.3)	59.5 (± 16.8)	** < 0.001**
Male	42 (82.4)	16 (45.7)	58 (67.4)	** < 0.001**
Length of stay	25.96 (± 35)	14.69 (± 14.2)	21.37 (± 28.9)	**0.043**
Number of Death	-	5 (14.3)	5 (5.8)	**0.005**
spinal cord injury	8 (15.7)	3 (8.6)	11 (12.8)	0.162
- Paraplegia	3 (5.9)	-	3 (3.5)	0.144
- Tetraplegia	5 (9.8)	3 (8.6)	8 (9.3)	0.847
Sensory defects	5 (9.8)	1 (2.9)	6 (7.0)	0.214
ICU stay	34 (66.7)	22 (62.9)	56 (65.1)	0.716
Ankylosing spondylitis	1 (2)	6 (17.1)	7 (8.1)	**0.011**
Osteoporosis	2 (3.9)	9 (25.7)	11 (12.8)	**0.003**

Table 2 summarizes injury mechanisms and associated injuries. Multivehicle accidents represented the predominant trauma mechanism, significantly affecting the < 65 years group. Falls from stairs were notably more frequent in the ≥ 65 years group (20% vs. 3.9%; p < 0.05). Other causes, including low-height falls (< 2 m), occurred frequently overall (30.2%) but without significant differences between groups. Additional injuries were common (overall prevalence 83.7%), significantly higher in the < 65 years group (90.2% vs. 74.3%; p < 0.05). Thoracic and abdominal injuries occurred significantly more often in the < 65 years group, whereas incidences of head, extremity, and other unspecified injuries showed no significant differences between groups.

**Table 2:** Injury mechanism and additional injuries

Injury mechanism and additional injuries	Patients < 65 N = 55	Patients ≥ 65 N = 31	Total N = 86	p-value
		N (%) or		
Traffic accident	28 (54.9)	8 (22.9)	36 (41.9)	**0.003**
Fall from height > 2 m	2 (3.9)	1 (2.9)	3 (3.5)	0.792
Fall of stairs	2 (3.9)	7 (20)	9 (10.5)	**0.017**
Sport related	4 (7.8)	3 (8.6)	7 (8.1)	0.903
Others (including falls from lower height < 2 m)	16 (31.4)	16 (45.7)	32 (37.2)	0.176
Total number of patients with additional injuries	46 (90.2)	26 (74.3)	72 (83.7)	**0.050**
-Head injury	14 (27.5)	5 (14.3)	19 (22.1)	0.148
-Thoracal injury	30 (58.8)	10 (28.5)	40 (46.5)	**0.006**
-Abdominal injury	15 (29.4)	2 (5.7)	17 (19.8)	**0.007**
-Extremity injury	24 (47.1)	14 (40)	38 (44.2)	0.517
-Other	16 (31.4)	10 (28.6)	26 (30.2)	0.781

In total, 343 spinal fractures were documented (Table 3), with more fractures observed in the < 65 years group (221 vs. 122); however, this difference was not statistically significant (p = 0.051). The mean fracture count per spinal segment per patient differed significantly only in the semi-rigid region, with more fractures in the < 65 years group (1.7 ± 1.4 vs. 1.1 ± 1.2; p = 0.042). In the < 65 years group, fractures occurred most frequently in semi-rigid (1.7 ± 1.4) followed by junctional regions (1.4 ± 1.2). In the ≥ 65 years group, junctional fractures predominated (1.5 ± 0.9), followed by semi-rigid (1.1 ± 1.2). AO type A fractures were most common overall (2.8 ± 2.1 fractures/patient), followed by type B (0.7 ± 1.1), type C (0.2 ± 0.5), and type F (0.2 ± 0.5). Multiple type B and C fractures occurred in 18.6% and 4.7% of patients, respectively. A significant difference by age was found only for AO type A fractures (< 65 years: 3.2 ± 2.0 vs. ≥ 65 years: 2.2 ± 2.1; p = 0.041).

**Table 3:** Fracture demographics

Fracture demographics	Patients < 65 N = 55	Patients ≥ 65 N = 31	Total N = 86	p-value
		N (%) or	Mean (± SD)	
Total number of fractures	221	122	343	0.051
Fractures per patient				
-Fractures in junctional segment	1.4 (± 1.2)	1.5 (± 0.9)	1.5 (± 1.1)	0.638
-Fractures mobile segment	1.3 (± 1.1)	0.9 (± 1.1)	1.1 (± 1.1)	0.122
-Fractures semi rigid segment	1.7 (± 1.4)	1.1 (± 1.2)	1.4 (± 1.4)	**0.042**
- Type A Fractures	3.2 (± 2)	2.2 (± 2.1)	2.8 (± 2.1)	**0.041**
- Type B Fractures	0.7 (± 0.1)	0.9 (± 1.4)	0.7 (± 1.1)	0.383
- Type C Fractures	0.2 (± 0.6)	0.2 (± 0.5)	0.2 (± 0.5)	0.558
- Type F fractures	0.2 (± 5.2)	0.1 (± 0.5)	0.2 (± 0.5)	0.534
Cases with multiple Type B fractures	10 (18.2)	6 (19.4)	16 (18.6)	0.773
Cases with multiple Type C fractures	3 (5.5)	1 (3.2)	4 (4.7)	0.513

Overall, surgical treatment was performed in 76.5% of patients (Table 4). Duration of surgery tended to be longer in the ≥ 65 years group, although this difference was not statistically significant (p = 0.095).

**Table 4:** Surgical treatment

Surgical treatment	Patients < 65 N = 55	Patients ≥ 65 N = 31	Total N = 86	p-value
		N (%) or	Mean (± SD)	
Non-surgical treatment	12 (23.5)	7 (20)	19 (22.1)	0.698
Single staged surgical treatment	39 (76.5)	27 (77.1)	66 (76.7)	0.942
Duration of single staged surgical treatment (min)	143.87 (± 67.4)	193.81 (± 140.5)	164.3 (± 105.6)	0.095

Table 5 summarizes complications and surgical revisions. Medical complications occurred in 14 patients, with pneumonia being the most frequent, particularly affecting the ≥ 65 years group (62.5% vs. 33.3%). Sepsis (28.6%), multiorgan failure (14.3%), and pulmonary embolism (7.1%) were rare, without significant age differences. Surgical revisions occurred in 16 cases, comprising hardware revisions (41.7%) and wound revisions (58.3%), with no significant differences observed between the two age groups.

**Table 5:** Complication and revision

	Patients < 65 N = 55	Patients ≥ 65 N = 31	Total N = 86	p-value
		N (%) or	Mean (± SD)	
Total number of medical complications	6	8	14	0.171
-Pneumonia	2 (33.3)	5 (62.5)	7 (50)	0.185
-Sepsis	2 (33.3)	2 (26)	4 (28.6)	0.698
-Acute pulmonary embolism	1 (16.6)	0	1 (7.1)	0.405
-Multiorgan failure	1 (16.6)	1 (12.5)	2 (14.3)	0.786
Total number of revisions	6	6	12	0.479
- Hardware	2 (33.3)	3 (50)	5 (41.7)	0.365
- Wound	4 (66.6)	3 (50)	7 (58.3)	0.903

## Discussion

Noncontinuous multilevel fractures (NMF) of the spine represent a complex injury group. They are challenging to diagnose, often overlooked, and difficult to treat due to skip-level injuries and frequent associated trauma. In our cohort, we identified 343 fractures among 86 patients, with a higher incidence in patients < 65 years compared to those ≥ 65 years, although this difference narrowly missed statistical significance (p = 0.051). Spinal fracture morphology is strongly influenced by the energy of the causative trauma, with higher kinetic energy consistently associated with increased fracture severity and quantity [9–12]. Therefore, the higher incidence observed in the < 65 years group is likely due to differences in trauma mechanisms.

Previous studies have identified the thoracic spine, lumbar spine, and thoracolumbar junction as the most commonly affected regions in NMF [1, 5, 8]. Wang et al. highlighted a higher frequency of fractures at the cervical-thoracic junction among patients < 65 years [5]. Our results align with these findings, demonstrating most fractures in the junctional region overall, followed by the semi-rigid and mobile segments. Although significant intergroup differences emerged only within the semi-rigid region, fracture distribution varied notably by age group, confirming previous observations by Wang et al. [5]. The most common fracture type across both age groups was AO type A, followed by types B and C. The frequency of type A fractures was significantly higher in the < 65 years group (3.2 ± 2 vs. 2.2 ± 2.1; p = 0.041). Although multiple type B or C fractures were more frequent in the < 65 years group, this did not reach statistical significance. These results support previous literature linking greater fracture severity to higher kinetic energy [9–12]. Our analysis further confirmed the vulnerability of junctional and semi-rigid spinal segments to trauma. While high-energy events such as motor vehicle accidents (MVA) or falls from heights were common mechanisms, low-energy trauma—such as stair falls or low-height falls—also caused significant injury, particularly among patients ≥ 65 years, likely due to age-related osteoporosis. Osteoporosis significantly increases fracture risk, and dedicated osteoporotic fracture classifications underscore this vulnerability [3, 5]. Consistent with existing studies, MVAs predominated overall (41.9%) and were significantly more frequent in the < 65 years group, whereas stair falls were notably more prevalent in the ≥ 65 years group [2, 3].

The relationship between injury mechanism and spinal fracture location is established: cervical-thoracic fractures typically result from MVAs, whereas lumbar fractures are more frequently linked to falls [8–10]. Our findings reinforce the association between higher-energy trauma and an increased number of fractures and more severe fracture types, predominantly observed in the < 65 years group, despite not always reaching statistical significance. Associated injuries occurred in 83.7% of cases, with thoracic (46.5%) and extremity injuries (44.2%) most prevalent. Thoracic and abdominal injuries were significantly more common in the < 65 years group, reflecting the higher kinetic energy of trauma in younger populations. This aligns with existing evidence indicating that specific spinal regions correlate with distinct extra-spinal injuries; cervical spine fractures commonly co-occur with head injuries, whereas thoracic and lumbar fractures frequently coincide with rib and pelvic injuries, respectively [6, 8, 10].

Spinal cord injury (SCI) was present in 19.8% of patients, with tetraplegia most frequent. Contrary to previous literature, we observed no significant difference in SCI rates between age groups, although our SCI incidence was comparatively low, possibly due to a smaller cohort size [3, 5]. Nevertheless, this substantial rate of neurological impairment highlights the serious threat posed by NMF. Treatment strategies for NMF, like other spinal fractures, depend on fracture stability, neurological involvement, and patient-specific comorbidities [9, 10]. No standardized treatment algorithm currently exists. Surgical management occurred in 77.9% of cases, similar to findings by Wang et al. [5]. Revision surgery was infrequent overall. Surgical duration for initial procedures averaged 164.3 ± 105.6 min and 139.2 ± 64.7 min for revision surgeries. Although not statistically significant, patients ≥ 65 years exhibited a trend towards longer operative times, potentially reflecting increased surgical complexity due to osteoporosis or comorbidities. Notably, 22.1% of cases underwent non-surgical management, suggesting that certain NMF—especially type A fractures in semi-rigid regions—may not inherently lead to spinal instability. The overall complication rate was low (14 patients). Despite the lack of statistical significance, medical complications were more frequent in patients ≥ 65 years, which accounted for 50% of pneumonia cases and all mortality events. This emphasizes heightened vigilance for postoperative complications and mortality in elderly populations, aligning with previous literature [11, 12].

Several limitations warrant consideration. Firstly, our retrospective data spanning eight years encompassed evolving imaging protocols, perioperative management, and documentation practices, introducing potential heterogeneity and confounding factors. Secondly, focusing exclusively on NMF without a comparative single-level fracture cohort may introduce selection bias, influenced by referral patterns or surgical preferences. Moreover, in-hospital complications alone were recorded, potentially underreporting true complication rates. Lastly, some subgroups were too small to robustly analyze independent associations between comorbidities, fracture morphology, and neurological outcomes.

## Conclusion

Noncontinuous multilevel spinal fractures constitute a complex spinal injury, often associated with neurological deficits. Our findings demonstrate distinct trauma mechanisms between age groups, yet similar fracture morphologies. The < 65 years group experienced significantly more associated injuries, whereas pneumonia was predominant among patients ≥ 65 years. Although most patients required surgical management, we observed no differences between the cohorts. Overall, not all NMF may necessarily lead to spinal instability, requiring operative intervention. Despite similarities these results underscore the need for age-adapted diagnostic and perioperative management protocols. Future research should further evaluate age-specific treatment algorithms concerning functional outcomes, quality-of-life, and health-economic implications.

## Data Availability

The dataset used the current study is available from the corresponding author on reasonable request.
